# Programmed Death-Ligand 1 Expression Potentiates the Immune Modulatory Function Of Myeloid-Derived Suppressor Cells in Systemic Lupus Erythematosus

**DOI:** 10.3389/fimmu.2021.606024

**Published:** 2021-04-27

**Authors:** Min-Jung Park, Jin-Ah Baek, Jeong Won Choi, Se Gwang Jang, Da-Som Kim, Sung-Hwan Park, Mi-La Cho, Seung-Ki Kwok

**Affiliations:** ^1^ The Rheumatism Research Center, The Catholic University of Korea, Seoul, South Korea; ^2^ Division of Rheumatology, Department of Internal Medicine, Seoul St. Mary’s Hospital, College of Medicine, The Catholic University of Korea, Seoul, South Korea

**Keywords:** programmed death-ligand 1, myeloid-derived suppressor cells, systemic lupus erythematosus, cell therapy, regulatory B cells

## Abstract

Multiple studies have explored the potential role of programmed death-ligand 1 (PD-L1) as a mediator of Myeloid-derived suppressor cells (MDSCs) effects in various cancers. However, the role PD-L1 expression in MDSCs on autoimmune disease is still largely unknown.This study was undertaken to whether MDSC expressing PD-L1 have more potent immunoregulatory activity and control autoimmunity more effectively in two murine models of lupus (MRL/*lpr* mice and Roquin^san/san^ mice). The populations of MDSC were increased in peripheral blood of lupus patients. The mRNA levels of immunosuppressive molecules were profoundly decreased in MDSCs from lupus patients and mice. Co-culture with splenocytes showed that PD-L1 expressing MDSCs from control mice expand both Treg cells and regulatory B cells more potently. Infusion of PD-L1 expressing MDSCs reduced autoantibody levels and degree of proteinuria and improved renal pathology of two animal models of lupus. Moreover, PD-L1 expressing MDSCs therapy can suppress double negative (CD4-CD8-CD3+) T cells, the major pathogenic immune cells and follicular helper T cells in MRL/*lpr* mice, and podocyte damage. Our results indicate PD-L1 expressing MDSCs have more potent immunoregualtory activity and ameliorate autoimmunity more profoundly. These findings suggest PD-L1 expressing MDSCs be a promising therapeutic strategy targeting systemic autoimmune diseases.

## Introduction

Systemic lupus erythematosus (SLE, lupus) is an autoimmune disease characterized by the production of diverse autoantibodies and immune complex deposit in target tissues. It affects multiple organs and has significant morbidity and mortality. The etiology of SLE is multifactorial and includes contribution from the environment and genetic factors. The exact pathogenesis of lupus remains still poorly understood, although there has been much progress in researching the pathogenesis of lupus through the use of genetic variant identification, mouse models, gene expression studies, and epigenetic analyses, Nevertheless, it is evident that various immunologic abnormalities contribute to the pathogenesis of lupus (1, 2).

Myeloid-derived suppressor cells (MDSCs) are a heterogenous population of early myeloid progenitors, immature granulocytes, macrophages and dendritic cells. MDSCs are often generated and expanded under pathologic conditions such as tumor environment or chronic inflammation. They can play an important role because of their potent suppressive roles in the immune response. These cells express immunosuppressive factors such as arginase-1, inducible nitric oxide synthase (iNOS) or reactive oxygen species (ROS), which can induce the inactivation of various immune cells, especially T cells (3).

Based on the well-known immunomodulatory properties of MDSCs, it can be speculated that cell therapy with MDSCs might be a promising therapeutic strategy for treating lupus. In our previous research, we demonstrated for the first time that murine MDSCs induce the expansion of regulatory B (Breg) cells and ameliorate autoimmunity in the murine lupus model (4). However, since then there have been conflicting results regarding the role of MDSCs in lupus. One report showed that adoptive transfer of monocytic MDSCs shows therapeutic effect in lupus prone mice (5). In contrast, others reported that MDSCs have pathogenic role in lupus (6–9).

Therefore, we hypothesized that MDSCs from lupus might be dysfunctional and specific population of MDSCs could have more powerful immunoregulatory activity. In this study, we measured the population of MDSCs in lupus patients and investigated the levels of various immunosuppressive mediators in MDSCs originated from human lupus patients and lupus mice. We also examined the impact of programmed death-ligand 1 (PD-L1) expressing MDSCs on T cell proliferation and various effector T/B cell subsets *in vitro*. Additionally, we examined whether *in vivo* treatment with PD-L1 expressing MDSCs suppress autoimmune phenotype more profoundly in two animal models of lupus (Roquin ^san/san^ mice and MRL/*lpr* mice).

## Materials and Methods

### Animals

Female MRL/MpJ‐Fas*^lpr^*/J (MRL/*lpr*) and MRL/MpJ (+/+) mice were purchased from SLC Inc. (Japan) (n = 10 mice per group per experiment). Female Sanroque (Roquin^san/san^) mice between 15–20 weeks of age were purchased from the NIH (n = 5 mice per group per experiment) and subsequently bred under specific pathogen-free conditions in an animal facility with controlled humidity (55 ± 5%), light (12 h/12 h light/dark), and temperature (22 ± 1°C). The air in the facility passed through a HEPA filter system designed to exclude bacteria and viruses. Animals were fed mouse chow and tap water *ad libitum*. The protocols used in our study were approved by the Animal Care and Use Committee of the Catholic University of Korea (CUMC-2017-0038-02).

### *In Vivo* Models

All mice were randomized divided into three groups as follows: control (saline), PD-L1+ MDSC treatment (PD-L1(+) MDSC), and PD-L1- MDSC treatment (PD-L1(-) MDSC). In Roquin^san/san^ mice model, each mouse received a weekly intravenous (IV) injection of saline (n=5) or 5x10^5^ PD-L1+MDSC (n=5) or 5x10^5^ PD-L1-MDSC (n=5) at 14 and 15 weeks. One week after the last treatment, all mice of each groups (n=5) were sacrificed at 16 weeks. At that time, the serum, kidney and spleen were collected for analyses. In MRL/*lpr* model, each mice received a weekly IV injection of saline (n=10) or 1x10^6^ PD-L1+MDSC (n=10) or 1x10^6^ PD-L1-MDSC (n=10) from 10 to 16 weeks of age. One week after the last treatment, all mice of each groups (n=10) were sacrificed at 17 weeks. At that time, the serum, kidney and spleen were collected for analyses.

### *In Vitro* Generation of MDSC

We isolated Bone marrow cells (BMCs) from the tibias and femurs of the mice by flushing the bone marrow cavity with Minimum Essential Medium (α-MEM; Invitrogen). BMCs were cultured in 6-well plate at 1 × 10^6^ cells/mL in complete medium supplemented with recombinant GM-CSF (20 ng/mL, PeproTech, Rocky Hill, NJ, USA) for 3 days. The cultured cells were harvested and stained with anti-CD11c FITC, anti-CD11b PerCP5.5, anti-Gr-1 APC, and anti-PD-L1 PE Ab (BD Biosciences) following FcR blocking (BD Biosciences). CD11c-CD11b+Gr-1+PD-L1+ and CD11c-CD11b+ Gr-1+PD-L1-MDSC subsets were sorted using a Beckman Coulter MoFlo XDP. The purity of the sorted PD-L1+MDSC and PD-L1MDSC was > 95%.

### Characterization of Human MDSC

Peripheral blood mononuclear cells (PBMCs) from SLE patients were stained with anti-Lineage, anti-HLA-DR, anti-CD11b, anti-CD33 anti-CD14, anti-CD15, anti-Arginase-1, anti-PD-L1 and anti-IL-10 Ab (Thermo fisher Scientific). Human total MDSCs analyzed as Lin^−^ HLA-DR^−^ CD11b^+^CD33^+^ population. Informed consent was obtained from all participants according to the principles of the Declaration of Helsinki. This trial was approved by the Institutional Review Board of Seoul St. Mary’s Hospital of the Catholic University of Korea (approval number: KC16CISF0365).

### T Cell Suppression Assay

PD-L1+ or PD-L1- MDSCs were isolated from MRL/MpJ mice. CD4+T cells were isolated from spleen of MRL/*lpr* mice by using the CD4+ T cell microbeads. Isolated PD-L1+ or PD-L1- MDSCs were cocultured with Cell Trace-violet -labelled CD4+ T cells (MDSC:T cell ratio 1:1, 1/2:1, 1/5:1). CD4+T cells were stimulated by 1 μg/ml of anti-CD3/CD28 (eBioscience, San Diego, CA, USA) and 30U/ml of recombinant IL-2 (R&D Systems, Minneapolis, MN, USA). After 5 days, proliferation of T cells was analyzed using FACS LSRFortessa cytometer (BD Pharmingen, San Jose, CA, USA).

### Co-Culture Experiments

PD-L1+ or PD-L1- MDSCs, CD4+T cells, splenocytes were isolated from normal C57BL/6 mice. Isolated PD-L1+ or PD-L1- MDSCs were cocultured with CD4+ T cells (MDSC:T cell ratio 1:1, 1/5:1, 1/20:1). Isolated PD-L1+ or PD-L1- MDSCs were cocultured with CD4+ T cells (MDSC:T cell ratio 1:1, 1/5:1, 1/20:1) under T cell activation condition (CD3/CD28 stimulation). For B10 analysis, PD-L1+ or PD-L1- MDSCs were cocultured with splenocyte (MDSC:splenocytes ratio 1:1, 1/5:1, 1/20:1) under LPS (100ng/ml) stimulation.

### FACS Analysis

Mononuclear cells were stained with various combinations of the following fluorescence-conjugated antibodies: CD19, CD1d, CD5, CD21, CD23, CD4, CD8, CD25, CD95, GL-7, CD138, PD-1, CXCR5 and B220 (BD Biosciences, San Diego, CA, USA). These cells were also intracellularly stained with the following antibodies: IFN-r, IL-4, IL-2, IL-10, IL-17 and Foxp3 (all eBioscience, San Diego, CA, USA). Before intracellular staining, the cells were restimulated for 4 hr with 25 ng/mL PMA (Sigma-Aldrich) and 250 ng/mL ionomycin (Sigma-Aldrich) in the presence of GolgiStop (BD Biosciences). Intracellular staining was performed using an intracellular staining kit (eBioscience) according to the manufacturer’s protocol. Flow cytometry was performed using a FACSCalibur and FACS LSRFortessa cytometer (BD Biosciences).

### Measurement of Cytokine Levels

The concentrations of IFN-γ, IL-10 and IL-17 in culture supernatants were measured by using a sandwich enzyme-linked immunosorbent assay (ELISA Duoset; R&D Systems, Lille, France).

### Measurement of IgG Titers and Anti-Double-Stranded DNA (Anti-dsDNA) Antibodies

Serum levels of IgG2a antibodies were measured using a commercially available ELISA kit (Bethyl Laboratories). The IgG anti-dsDNA antibody was measured by ELISA using serum samples diluted at 1:500 and alkaline phosphatase (AP)-conjugated goat-anti mouse IgG, secondary antibody at a 1:1,000 dilution.

### Measurement of Albumin

Urine albumin and creatinine concentrations were measured using a mouse albumin ELISA assay (Bethyl Laboratories, Montgomery, TX, USA) and a creatinine assay (R&D systems), according to the manufacturer’s directions.

### Assessment of Histopathology of Nephritis

We embedded formalin-fixed tissues in paraffin and sectioned them into 5 μm sections. We stained them with hematoxylin (Sigma-Aldrich) and eosin (Muto Pure Chemical Co., Ltd, Tokyo, Japan) for histological examination (H&E). Kidney tissues were stained with periodic acid–Schiff (PAS; Sigma-Aldrich). Renal pathology including glomerular pathology, tubular injury, and perivascular cell accumulation was evaluated as described previously (10) by 2 investigators in a blinded manner.

### Real-Time Polymerase Chain Reaction (PCR)

Messenger RNA (mRNA) was extracted using the TRI Reagent (Molecular Research Center, Inc. Cincinnati, OH, USA) according to the manufacturer’s instructions. Complementary DNA was synthesized using a SuperScript Reverse Transcription system (Takara Bio Inc., Otsu, Japan). A LightCycler 2.0 instrument (software version 4.0; Roche Diagnostics, Mannheim, Germany) was used for PCR amplification. All reactions were performed using the LightCycler FastStart DNA Master SYBR Green I mix (Takara Bio Inc.), following the manufacturer’s instructions. Primer sequences are described in the **Table S1**. All mRNA levels were normalized to that of β-actin.

### Confocal Microscopy and Immunostaining

Mouse and human kidney tissue cryosections (7 μm thick) were fixed in 4% (v/v) paraformaldehyde and stained using fluorescein isothiocyanate (FITC)-, phycoerythrin (PE)-, PerCP-Cy5.5-, or allophycocyanin -conjugated monoclonal antibodies to mouse nephrin (PROGEN), WT-1 (Abcam), C3, IgG and human CD11b (Abcam), CD33 (Abcam), DAPI (Invitrogen). After incubation overnight at 4 °C, stained sections were visualized by confocal microscopy (LSM 510 Meta; Zeiss, Göttingen, Germany).

### Statistical Analysis

Statistical analyses were performed using GraphPad Prism version 8.0. The experimental data are presented as mean ± SEM. Comparisons of numerical data between two groups were performed by Student’s *t*-tests or Mann-Whitney U-test. Comparisons between multiple groups were analyzed by one‐way analysis of variance. *P* values less than 0.05 (2-tailed) were considered significant.

## Results

### PD-L1 Expressing MDSCS Have More Potent *In Vitro* Immunoregulatory Activity in Mice

**Figure 1:**
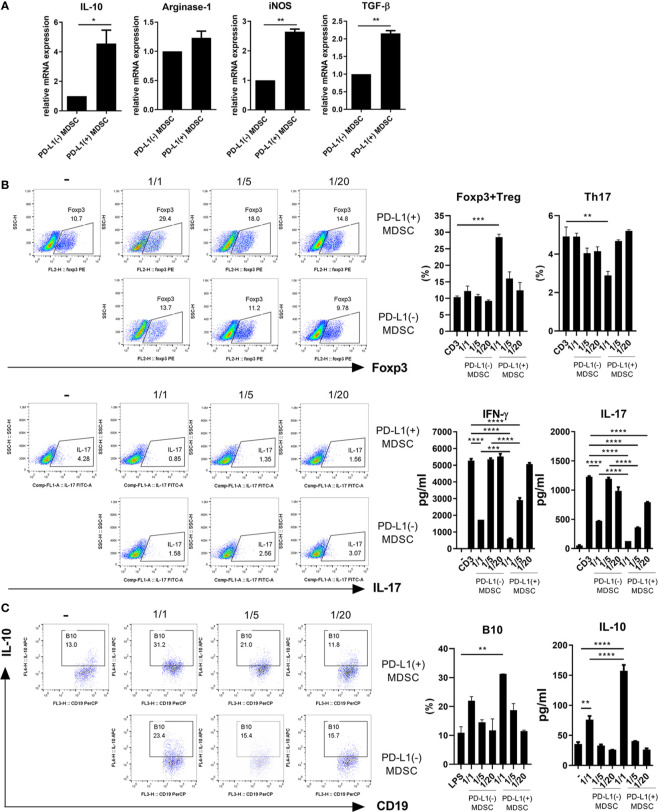
PD-L1 expressing MDSCs have stronger *in vitro* immunoregulatory activity in mice. **(A)** MDSCs were obtained from C57BL/6 mice as described in Materials and Methods section. MDSCs sorted into PD-L1+ and PD-L1− sub-populations using a cell sorter. **(A)** The mRNA levels of IL-10, arginase-1, iNOS and TGF-β in PD-L1 positive or negative MDSCs were determined using real-time PCR. **(B)** CD4+T cells (5 × 10^5^) were cultured with PD-L1 positive or negative MDSCs in the presence of anti-CD3 (0.5 μ/ml). After 3 days, cells were analyzed for Treg (CD4+CD25+Foxp3+) and Th17 (CD4+IL-17+) cells. The representative flow cytometric plot is shown. The percentage of each cell population among CD4+ T cells are shown in the right upper panel. The concentrations of IL-17 and IFN-γ in culture supernatant were measured by ELISA. **(C)** Splenocytes (5 × 10^5^) were cultured with PD-L1 positive or negative MDSCs in the presence of LPS (100 ng/ml). After 3 days, cells were analyzed for IL-10 producing B10 cells (CD19+CD1d+CD5+ cells) by flow cytometry. The percentage of each cell population are shown in the right panel. The concentrations of IL-10 in supernatant were measured by ELISA. Data are expressed as the mean ± SEM. Data are representative of three independent experiments (*P < 0.05, **P < 0.01, ***P < 0.001, ****P < 0.0001).

We hypothesized that PD-L1 positive MDSCs could have potent immunoregulatory activity *in vitro*. In an attempt to test whether PD-L1 positive MDSCs could be more immunosuppressive, MDSCs were generated from bone marrow cells from C57BL/6 mice as described in Materials and Method section. We first analyzed the mRNA levels of crucial immunoregulatory mediators like IL-10, Arginase-1, iNOS, and TGF-β using real-time PCR. As shown in **Figure 1A**, the mRNA levels of IL-10, iNOS, and TGF-β were significantly increased in MDSCs from PD-L1 positive MDSCs compared to PD-L1 negative MDSCs. We next cultured CD4+ T cells with medium alone or graded dose of PD-L1 positive or negative MDSCs under anti-CD3 stimulation and checked the proinflammatory IL-17 producing CD4+ T cells (Th17 cells) and anti-inflammatory CD4+CD25+Foxp3+ regulatory T (Treg) cells. The results showed that *in vitro* treatment with PD-L1 positive MDSCs more profoundly decreased Th17 cells and increased Treg cells compared to PD-L1 negative MDSC (**Figure 1B**). The levels of IL-17 and IFN- γ, the pro-inflammatory cytokines that are implicated with the pathogenesis of lupus, were decreased in culture supernatant of PD-L1 positive MDSCs and CD4+T cells (**Figure 1B**). We also cultured spleen cells with medium alone or graded dose of PD-L1 positive or negative MDSCs under LPS stimulation condition and the IL-10 producing B10 cells, which is considered as regulatory B (Breg) cells, were analyzed. The results showed that *in vitro* treatment with PD-L1 positive MDSCs more profoundly increased Breg cells compared to PD-L1 negative MDSC (**Figure 1C**). The levels of IL-10, the prototype anti-inflammatory cytokine, are increased in culture supernatant of PD-L1 positive MDSCs and spleen cells (**Figure 1C**).

### MDSCs From Lupus Mice Could Be Dysfunctional and PD-L1 Positive MDSCs From Control Mice Have More Potent *In Vitro* Immunoregulatory Activity Compared to Those From Lupus Mice

**Figure 2:**
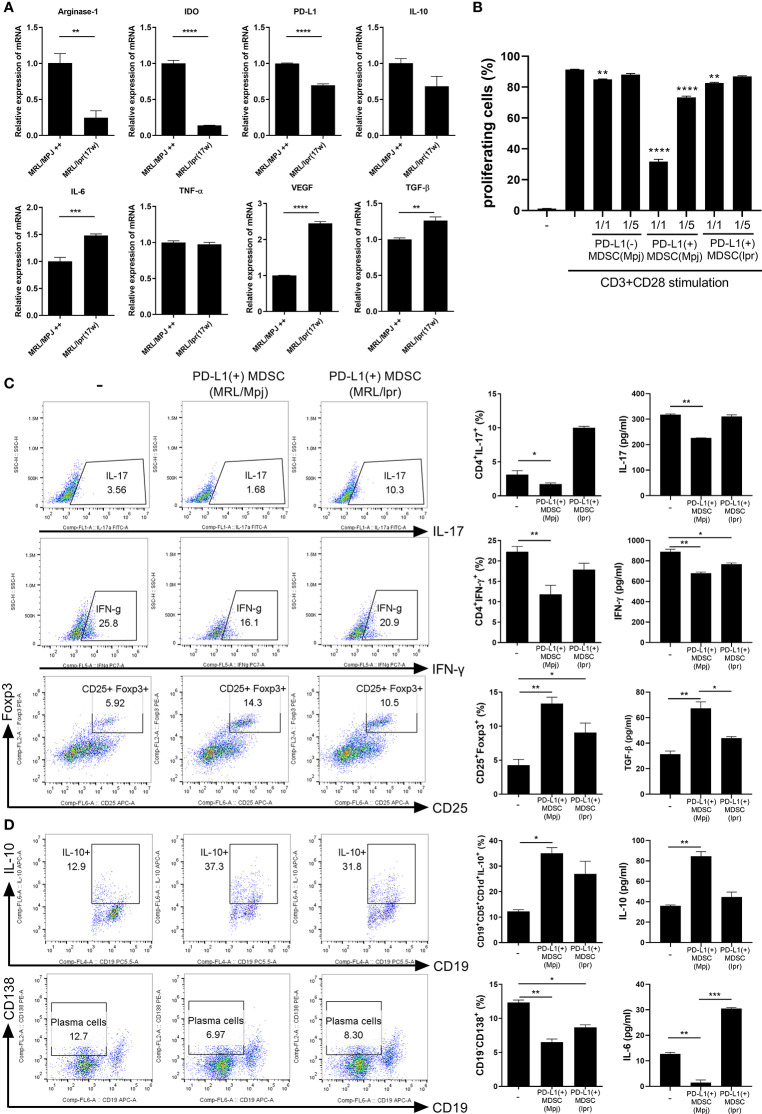
PD-L1 expressing MDSCs from control mice shows more powerful immune regulatory activity *in vitro*. **(A)** PD-L1 positive MDSCs from lupus prone MRL/*lpr* mice and MLR/MpJ (control mice) were isolated. The mRNA levels of pro or anti-inflammatory molecules were determined. **(B)** CD4+ T cells (5 × 10^5^) from MLR/*lpr* mice were labeled with Cell trace violet, and cultured with/without PD-L1 positive or negative MDSCs (1:1, 5:1 ratio). The percentage of proliferating CD4+ cells were analyzed. **(C)** CD4+ T cells from MLR/*lpr* mice were cultured with PD-L1 positive MDSCs from MRL/*lpr* mice and MLR/MpJ (1:1 ratio) in the presence of anti-CD3 (0.5 μ/ml). After 3 days, cells were analyzed for Th17, Th1 and Treg cells by flow cytometry. The percentage of each cell population are shown in the right panel. The concentrations of IL-17, IFN- γ and TGF-β in supernatant were measured by ELISA. **(D)** Splenocytes from MLR/*lpr* mice were cultured with PD-L1 positive MDSCs (1:1 ratio) in the presence of LPS (100 ng/ml). After 3 days, cells were analyzed for IL-10 producing B10 cells and plasma cells (CD19-CD138+) by flow cytometry. The percentage of each cell population are shown in the right panel. The concentrations of IL-10 and IL-6 in supernatant were measured. Data are expressed as the mean ± SEM. Data are representative of three independent experiments (*P < 0.05, **P < 0.01, ***P < 0.001, ****P < 0.005).

In an attempt to verify that MDSCs from lupus could be dysfunctional, we analyzed the mRNA levels of various immunosuppressive factors in MDSCs generated from bone marrow cells from lupus mice (17 weeks old MRL/*lpr* mice) and control mice (MRL/MpJ mice). The mRNA levels of arginase-1, IDO, PD-L1 and IL-10 were profoundly decreased in MDSCs from MRL/*lpr* mice compared to MDSCs form control mice (**Figure 2A**). However, the mRNA levels of pro-inflammatory cytokines like IL-6 and VEGF was significantly increased in MDSCs from MRL/*lpr* mice (**Figure 2A**). We compared *in vitro* immunoregulatory activity of MDSCs from control or lupus mice and the results showed that MDSCs from lupus mice inhibit CD4+ T cell proliferation weakly (data not shown). These findings suggest that MDSCs from lupus mice would be dysfunctional like one previous report (11). We also compared *in vitro* immunoregulatory activity of three groups of MDSCs (PD-L1 positive or negative MDSCs from control mice, PD-L1 positive MDSCs from lupus mice). As expected, PD-L1 positive MDSCs from the control mice more markedly inhibit CD4+ T cell proliferation. To our surprise, both PD-L1 positive MDSCs from lupus mice and PD-L1 negative MSDSs from control mice weakly inhibit CD4+ T cell proliferation (**Figure 2B** and **Figure S1**). We next investigated the effect of PD-L1 positive MDSCs on effector T/B cell population *in vitro*. When the CD4+ T cells from lupus mice were cultured with PD-L1 positive MDSCs from lupus and/or control mice, only PD-L1 positive MDSCs obtained from control mice markedly inhibited proinflammatory Th1, Th17 cells and reciprocally induced Foxp3+ Treg cells. The levels of IL-17 and IFN- γ in coculture supernatant were significantly decreased and levels of TGF-β, the prototypic anti-inflammatory cytokine, were increased only in PD-L1 positive MDSCs from control mice treated group (**Figure 2C**). Furthermore, when the spleen cells from lupus mice (MRL/*lpr* mice) were cultured with PD-L1 positive MDSCs, only PD-L1 positive MDSCs form control mice remarkably suppressed plasma cell (CD19-CD138+) population and expanded IL-10 producing B 10 cell (CD19+CD5+CD1d+) (**Figure 2D**).

### Effect of Cell Therapy With PD-L1 Expressing MDSCs on the Lupus-Like Phenotype of Roquin^san/san^ Mice

**Figure 3:**
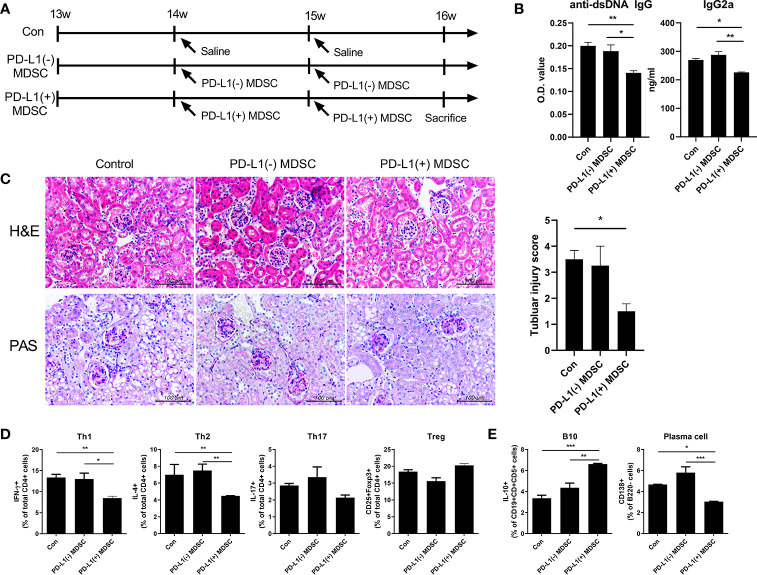
*In vivo* treatment with PD-L1 positive MDSCs shows better therapeutic effects in lupus prone Roquin^san/san^ mice. **(A)** 13-week-old Roquin^san/san^ mice (n=5 in each group) were treated intravenously with 1 × 10^6^ PD-L1 positive and/or negative MDSCs from C57BL/6 mice or vehicle (saline) once a week for 2 weeks. One week after the last treatment, Roquin^san/san^ mice was sacrificed. **(B)** Serum levels of IgG2a and anti-dsDNA IgG in Roquin^san/san^ mice treated with PD-L1 positive or negative MDSCs or saline, as determined by ELISA. **(C)** Representative images of hematoxylin and eosin (H&E) and PAS staining of the kidney sections are shown in the left panel. Tubular injury score is shown in the right panel. **(D)** Flow cytometric analysis shows the populations of CD4+IFN-γ T cells (Th1 cells), CD4+IL-4+ T cells (Th2 cells), CD4+IL-17+ T cells (Th17 cells), CD4+CD25+FOXP3+ cells (Treg cells) in spleen tissues from Roquin^san/san^ mice. Data are expressed as the mean ± SEM. Data are representative of 2 independent experiments. Relative bar charts are shown. **(E)** Flow cytometric analysis shows the populations of B220-CD138+ cells (plasma cells) and CD19+CD1d+CD5+IL-10+ cells (IL-10 producing B10 cells) in spleen tissues from Roquin^san/san^ mice. Relative bar charts are shown. (*P < 0.05, **P < 0.01, ***P < 0.001).

We examined whether *in vivo* treatment with PD-L1 expressing MDSCs might have more beneficial effects on renal histopathology and autoantibody production in one animal model of lupus, Roquin^san/san^ mice, in which the sanroque mutation disrupts a repressor of ICOS, an essential co-stimulatory molecule for follicular helper T cells (Tfh cells), resulting in excessive formation of Tfh cells and germinal centers (GC) (12, 13). We initiated a therapeutic protocol consisting of intravenous infusion of 1 × 10^6^ PD-L1 positive or negative MDSCs obtained from C57BL/6 mice or an equal volume of saline to mice beginning at 13 weeks of age. We administered this once weekly for two weeks. One week after the last injection, the mice were sacrificed (**Figure 3A**). As shown in **Figure 3B**, serum concentrations of anti-dsDNA IgG and IgG2a markedly decreased following treatment with PD-L1 positive MDSCs whereas treatment with PD-L1 negative MDSCs did not have any effect (**Figure 3B**). Histologic examination revealed that Roquin^san/san^ mice treated with PD-L1 positive MDSCs showed remarkably decreased tubular injury score compared to saline or PD-L1 negative MDSCs (**Figure 3C**). However, there were no significant difference in glomerular pathology score among the three groups (data not shown). We also examined the effects of *in vivo* treatment with PD-L1 positive or negative MDSCs on various T/B cell subsets in spleens of Roquin^san/san^ mice. As shown in **Figures 3D, E** and **Figure S2**, infusion of PD-L1 positive MDSC profoundly decreased Th1 (CD4+ IFN-γ +) cells, the proinflammatory T cell subsets and plasma cell (CD138+B220-), but increased Breg (CD19+CD1d+CD5+IL-10+) cells compared to PBS or PD-L1 negative MDSCs. These results suggest that *in vivo* treatment with PD-L1 positive MDSCs more profoundly ameliorates the lupus-like phenotype of Roquin^san/san^ mice.

### PD-L1 Expressing MDSCs Have More Beneficial Impact on Not Only the Autoimmune Phenotype but also Podocyte Injury in MRL/*lpr* Mice

**Figure 4:**
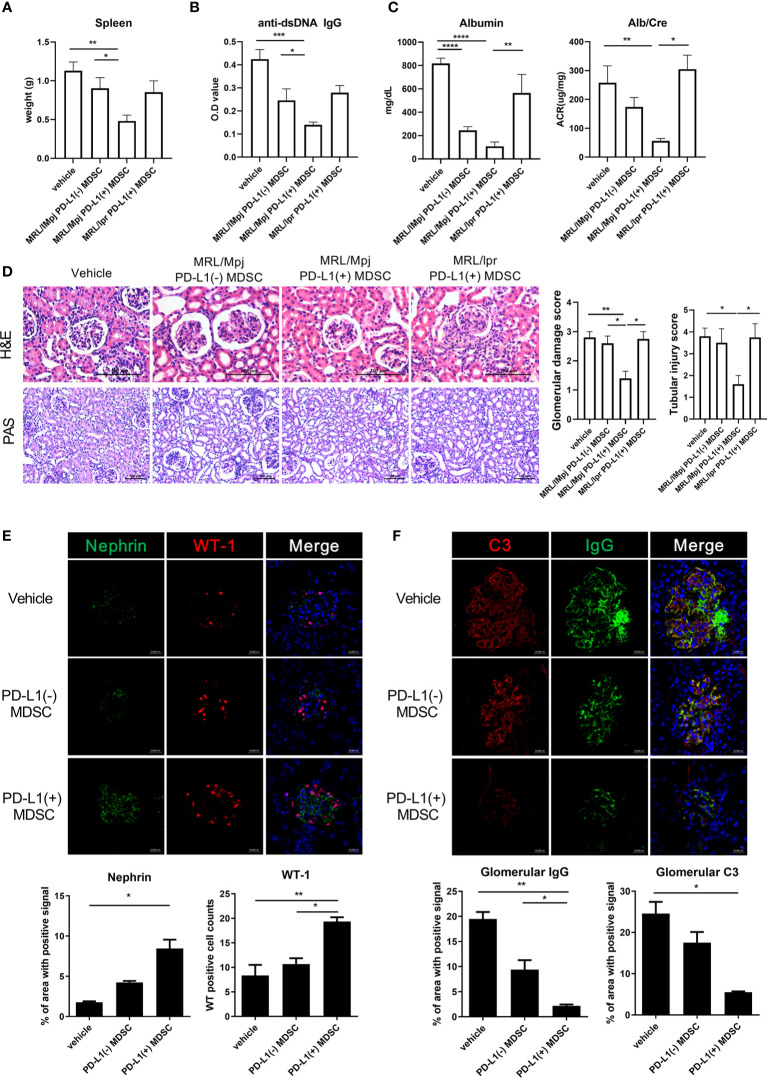
*In vivo* treatment with PD-L1 positive MDSCs shows better therapeutic effects in MRL/*lpr* mice. **(A)** Ten-week-old MRL/*lpr* mice (n=10 in each group) were treated intravenously with 1 × 10^6^ PD-L1 positive and/or negative MDSCs from MRL/MpJ mice or 1 × 10^6^ PD-L1 positive MDSCs from MRL/*lpr* mice once weekly for six weeks. One week after the last treatment, mice were sacrificed. Spleen weights are shown. **(B)** Serum levels of anti-dsDNA IgG were determined by ELISA. **(C)** Urinary albumin and Creatinine (Cr) levels were measured and Albumin/Cr ratio is shown. **(D)** Representative images of H&E and PAS staining of the kidney sections are shown in the left panel. Tubular injury and glomerular damage score is shown. **(E)** Representative confocal microscopic images of kidney sections are shown. Cells were stained with fluorescence-tagged antibodies to identify podocyte markers including nephrin (green) and Wilms tumor protein (WT-1) (red). Nephrin signal positive area were calculated using image J software and the mean values are presented. The cells showing positive WT-1 signal were enumerated and the mean values are presented. **(F)** Cells were stained with antibodies to identify C3 (red) and IgG (green). IgG and/or C3 signal positive area were calculated and the mean values are presented. Data are expressed as the mean ± SEM. Data are representative of 2 independent experiments [*p < 0.05, **p < 0.01, ***p < 0.001, ****p < 0.0001 versus control (vehicle)].

In order to investigate the therapeutic impact of PD-L1 positive MDSCs on other lupus-prone mice (MRL/*lpr* mice), MRL/*lpr* mice at the age of 8 weeks were intravenous injected with 1 × 10^6^ PD-L1 positive or negative MDSCs obtained from MRL/MpJ mice or 1 × 10^6^ PD-L1 positive MRL/*lpr* mice an equal volume of saline, which was administered once weekly for six weeks. One week after the last injection, the mice were sacrificed. As shown in **Figure 4A**, the weight of spleen was significantly lighter in mice treated with PD-L1 positive MDSCs compared to PD-L1 negative MDSCs or saline. Serum levels of anti-dsDNA IgG was significantly lower in mice treated with PD-L1 positive MDSCs from MRL/MpJ mice (**Figure 4B**). The degree of proteinuria, which was identified as urinary albumin level or albumin/Creatinine ratio, decreased profoundly following treatment with PD-L1 positive MDSCs from MRL/MpJ mice (**Figure 4C**). We also evaluated histologically kidney tissues of MRL/*lpr* mice treated with PD-L1 positive or negative MDSCs or saline. The results showed that only *in vivo* infusion of PD-L1 positive MDSCs from MRL/MpJ mice remarkably decreased glomerular pathology score and tubular injury score (**Figure 4D**). We also tested therapeutic efficacy of PD-L1 positive MDSCs from lupus mice (MRL/*lpr* mice). As shown in **Figures 4A–D**, PD-L1 positive MDSCs from lupus mice do not show meaningful therapeutic impact in animal model of lupus (MRL/lpr mice).

Podocytes are the essential component of glomerular filtration apparatus and critical for the maintenance of renal function (14). Podocyte injury is involved in the pathogenesis of lupus nephritis (15). Therefore, we investigated whether *in vivo* infusion of PD-L1 positive MDSCs could reverse podocyte injury in glomeruli of MRL/*lpr* mice. As shown in **Figure 4E**, the number of podocytes, defined as nephrin+WT-1+ cells by confocal microscopy, was significantly higher in mice treated with PD-L1 positive MDSCs. Immune complex deposit is the major pathologic feature in lupus nephritis. Therefore, we checked whether PD-L1 positive MDSCs could decrease the immune complex deposit in glomeruli of MRL/*lpr* mice. The results showed that MRL/*lpr* mice treated with PD-L1 expressing MDSCs showed significantly decreased immune complex deposit which was determined by IgG, C3 staining using confocal microscopy (**Figure 4F**). These findings suggest that PD-L1 expressing MDSCs attenuate podocyte injury as well as autoimmune phenotype in lupus prone mice (MRL/*lpr* mice).

### PD-L1 Expressing MDSCs Suppressed the Pro-Inflammatory Profile of T and B Cells and Expand Anti-Inflammamtory Treg Cells and Breg Cells in MRL/*lpr* Mice

**Figure 5:**
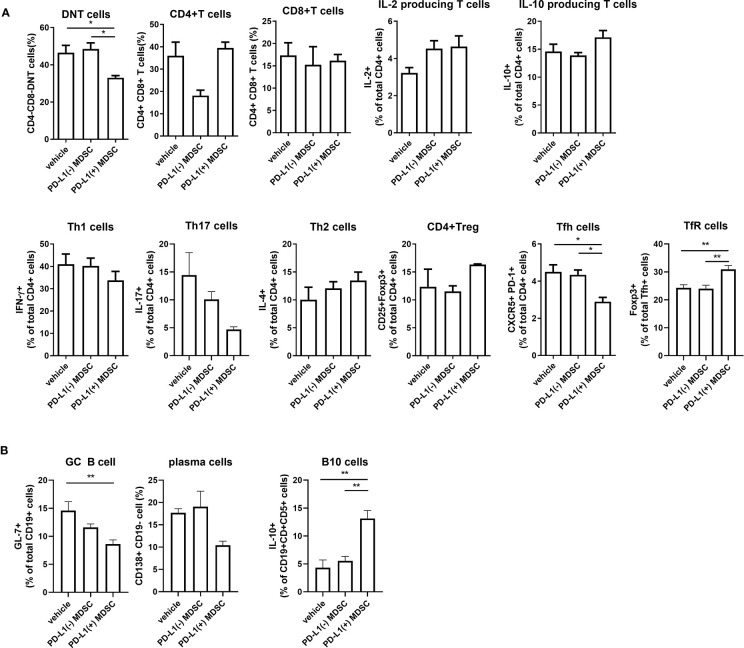
Cell therapy with PD-L1 positive MDSCs profoundly decreases pathogenic double negative T cells (DN T cells) in spleens of MRL/*lpr* mice. **(A)** Flow cytometric analysis shows the populations of DN T cells (CD4-CD8-CD3+ T cells), CD4+ T cells, CD8+ T cells, IL2+CD4+ T cells, CD4+IL-10+ T cells, CD4+IFN-γ T cells (Th1 cells), CD4+IL-17+ T cells (Th17 cells), CD4+IL-4+ T cells (Th2 cells), CD4+CD25+FOXP3+ cells (Treg cells), CD4+CXCR5+PD-1+ T cells (Tfh cells) and CD4+CXCR5+PD-1+ Foxp3+(TFR cells) in spleen tissues from MRL/*lpr* mice treated with PD-L1 positive and/or negative MDSCs obtained from MRL/MpJ mice. Relative bar charts are shown. **(B)** Flow cytometric analysis shows the populations of CD19+GL7+ cells (GC B cells), CD19-CD138+ cells (plasma cells) and CD19+CD1d+CD5+IL-10+ cells (IL-10 producing B10 cells) in spleen tissues from MRL/*lpr* mice. Relative bar charts are shown. Data are expressed as the mean ± SEM. Data are representative of three independent experiments [*P < 0.05, **P < 0.01 versus control (vehicle)].

Double-negative (DN) T cells are defined by the absence of CD4 and CD8 and the ability to produce proinflammatory cytokines like IFN-γ and IL-17 which has been linked to lupus pathogenesis both in human and mice (16). As shown in **Figure 5A**, administration of PD-L1 positive MDSCs dramatically decreased DN T cell population in spleens of MRL/*lpr* mice compared to PD-L1 negative MDSCs or saline. In addition to DNT cells, Tfh (CD4+CXCR5+PD-1+) cells, which is other proinflammatory T cell subsets, were decreased by treatment with PD-L1 positive MDSCs. In contrast, follicular regulatory T (CD4+FOXP3+CXCR5+PD-1+, Tfr) cells were increased by administration of PD-L1 positive MDSCs. We also analyzed the populations of effector B cells. The results showed that PD-L1 positive MDSCs profoundly deceased GC B cells (CD19+GL7+), but increased Breg (CD19+CD1d+CD5+IL-10+) cells (**Figure 5B**).

### Immunoregulatory Mediators Are Decreased in Myeloid-Derived Suppressor Cells (MDSCs) From Lupus Patients

**Figure 6:**
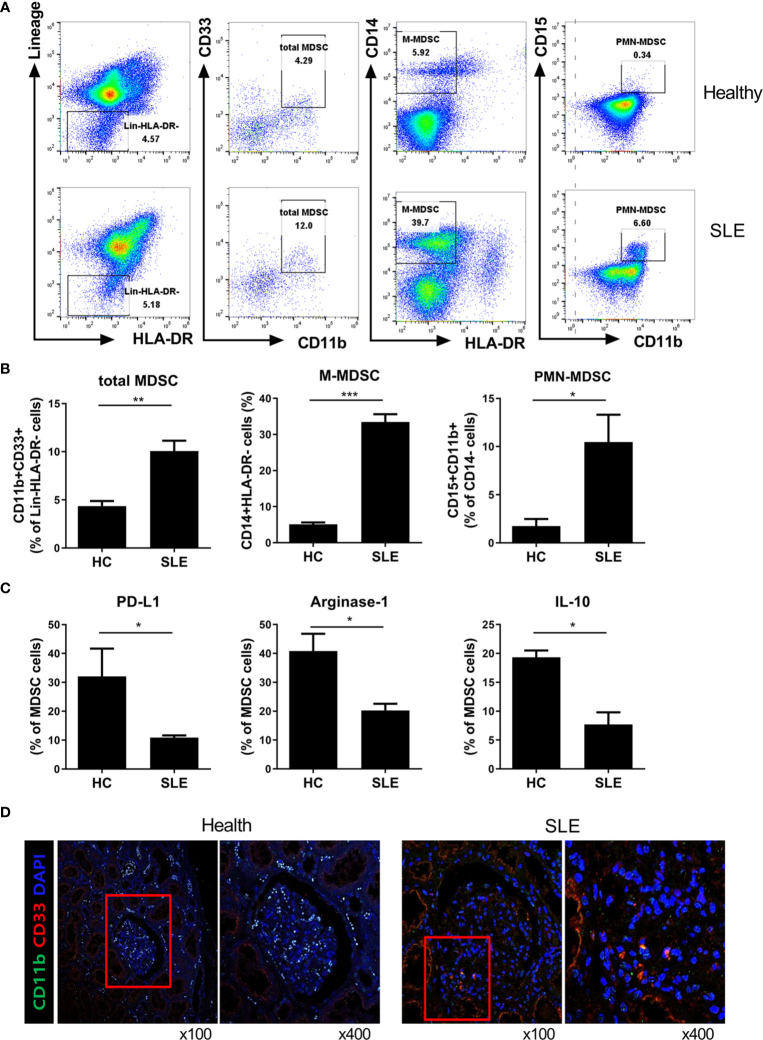
Characterization of MDSCs from SLE patients. **(A)** MDSCs subpopulations of human peripheral bloods was determined by flow cytometry in SLE patients and healthy controls (Total MDSC: HLA-DR-Lin-CD33+CD11b+ cells, M-MDSC: CD14+ HLA-DR- cells, PMN-MDSC: CD14-CD15+CD11b+ cells). Gating strategy of human MDSCs and representative flow cytometric plot is shown. **(B)** Relative bar graph indicates the frequency of MDSC subpopulation in healthy controls and SLE patients. **(C)** The expression of PD-L1, arginase-1, and IL-10 was determined by flow cytometry. The percentage of PD-L1, Arginase-1, and IL-10 expressing MDSCs is shown. **(D)** Representative confocal microscopy analysis for MDSC like cells in kidney of patients with lupus nephritis and healthy controls. Cells were stained with fluorescence-tagged antibodies to identify DAPI, CD11b (FITC) and CD33 (PE). CD11b+CD33+ cells, MDSC like cells, are located in kidney tissue sections, especially in patients with lupus nephritis (x100(left), x400(right), original magnification). (*P < 0.05, **P < 0.01, ***P < 0.001).

We finally examined the population of MDSCs in peripheral blood of patients with lupus and age, sex matched healthy controls. The MDSCs subset in the peripheral blood of SLE patients was analyzed by flow cytometry. Human total MDSCs (Lin-HLA-DR-CD11b+CD33+ cells), M-MDSC (HLA-DR-CD11b+CD14+ cells) and PMN-MDSC (CD11b+CD14+D15+ cells) were identified as defined previously (17). As shown in **Figures 6A, B**, the proportion of total MDSCs and two MDSC subsets were profoundly increased in peripheral blood of lupus patients compared to healthy controls as previously reported (7, 8). Interestingly, the mRNA levels of immunoregulatory molecules including PD-L1, arginase-1, and IL-10 were significantly decreased in lupus patients (**Figure 6C**). We next checked whether MDSCs are located in the kidney, which is the major target organ in lupus. The results showed that MDSCs like cells, defined as CD11b+CD33+ cells in our study, are located in kidney tissues of patients with lupus nephritis whereas not in healthy kidney area of patients with renal cell carcinoma (**Figure 6D**). Collectively, these results demonstrate that MDSCs are increased in lupus patient, but they show a defective immunoregulatory phenotype.

## Discussion

Myeloid-derived suppressor cells (MDSCs) are a heterogenous collection of myeloid cells composed of myeloid precursors, immature granulocytes, mononuclear macrophages, and dendritic cells. MDSCs are broadly characterized by CD11b+GR-1+ cells in mice and HLA-DR-CD11b+CD33+ cells in human and they typically divided into two subpopulations: polymorphonuclear MDSCs (PMN-MDSCs) and monocytic MDSCs (M-MDSCs). Increasing evidence suggests that MDSCs with immuosuppressive effect have therapeutic effects in various animal models of systemic autoimmune diseases including multiple sclerosis (18, 19), rheumatoid arthritis (20–22), inflammatory bowel disease (23), myasthenia gravis (24), type 1 diabetes (25) and uveitis (26). We reported for the first time that MDSCs generated from bone marrow of normal wild type mice induce the expansion of Breg cells by inducible nitric oxide synthase (iNOS) and ameliorate autoimmunity in the murine model of lupus (4). Contrary to our previous research, a few reports have shown that MDSCs might have pathogenic role in lupus both in human and mice (6, 7, 27). Theses discrepancies between studies might be related to the source of MDSCs or the method of MDSCs generation. However, we do not still know the exact role of MDSCs in lupus.

In an attempt to clarify the reason for such discordant results about the role of MDSCs in lupus, we hypothesized that MDSCs originated from lupus could be dysfunction like one previous report showing that the function PMN-MDSCs originated from (NZB X NZW) F1 lupus prone mice was impaired which was determined by T cell proliferation assay (11). In our study, MDSCs from another lupus mice (MRL/*lpr* mice) also showed defective *in vitro* immunoregulatory activity which was determined by T cell proliferation assay (data not shown). Therefore, it could be of utmost importance to find out specific population of MDSCs with more potent immunoregulatory activity.

Programmed death-ligand1 (PD-L1), which is a ligand for the inhibitory checkpoint molecule PD-1, is a 40kDa type 1 transmembrane protein that has been speculated to play a major role in suppressing the adaptive arm of immune system during particular events such as pregnancy, tissue allografts and autoimmune diseases. Noman et al. reported that blockade of PD-L1 under hypoxia enhanced MDSC-mediated T cell activation (28). Therefore, it can be speculated that PD-L1 expressing MDSCs might have more potent immunoregulatory activity and we found that the mRNA levels of diverse immunosuppressive molecules including IL-10, arginase-1, iNOS and TGF-β were significantly higher in PD-L1 positive MDSCs of wild type control C56BL/6 mice (**Figure 1A**). When MDSCs from control mice were culture with CD4+ T cells, *in vitro* treatment with PD-L1 positive MDSCs more profoundly decreased Th17 cells and increased Treg cells and Breg cells compared to PD-L1 negative MDSC (**Figures 1B, C**). These findings suggest that PD-L1 expressing MDSCs have more powerful immunosuppressive activity *in vitro*.

The mRNA levels of various immunosuppressive factors including arginase-1, IDO, PD-L1 and IL-10, which are known to mediate the immuoregulatory effects of MDSCs, were profoundly decreased in MDSCs from lupus prone mice (MRL/*lpr* mice) (**Figure 2A**). These findings indirectly support the previous report suggesting that the function of MDSCs from lupus prone mice could be impaired (11). We next compared *in vitro* immunoregulatory activity of three groups of MDSCs (PD-L1 positive or negative MDSCs from control mice, PD-L1 positive MDSCs from lupus mice). The results showed that PD-L1 positive MDSCs from control mice have more powerful immunoregulatory activity which was determined by T cell proliferation assay (**Figure 2B**). In addition, PD-L1 positive MDSCs from control mice more potently regulate effector T/B cell populations *in vitro* compared to PD-L1 positive MDSCs from lupus mice (**Figures 2C, D**).

In the present study, we also verified that PD-L1 positive MDSCs more profoundly suppress lupus phenotype including the degree of proteinuria, serum levels of immunoglobulins, autoantibodies (anti-dsDNA IgG) and histopathologic findings of kidneys compared to PD-L1 negative MDSCs in two animal models of lupus (Roquin ^san/san^ mice and MRL/*lpr* mice) (**Figures 3B, C** and **4B–D**).

Renal involvement is the most common and severe clinical manifestation seen in patients with lupus. It is characterized by immune complex deposition, inflammation, and scarring of glomeruli and interstitium (15). Podocytes are highly specialized cells that reside on the visceral side of the Bowman capsule and wrap around glomerular capillaries. They express markers such as synaptopodin, nephrin, podocin, and Wilms’ tumor protein (29). These molecules play important roles in maintaining filtration barrier integrity. Podocytes are the essential component of glomerular filtration apparatus and critical for the maintenance of renal function. So, it is evident that podocyte injury or loss can initiate glomerular damage, possibly leading to chronic kidney disease (CKD) (30). In addition, podocyte injury is involved in the pathogenic development of lupus nephritis (31). In this study, we demonstrated that *in vivo* infusion of MDSCs generated from control mice (MRL/MpJ mice) attenuate podocyte injury as well as immune complex deposit in kidneys of lupus prone mice (MRL/lpr mice) (**Figures 4E, F**). Our results is not consistent with one previous report showing that MDSCs generated from bone marrow cells of wild type mice induce podocyte injury through increasing reactive oxygen species *in vitro* (27). The discrepancy might be partially explained by the source of MDSCs or the difference between animal models although exact reason still remains to be determined. Further extensive research will be required to clarify this issue.

In the present study, we used MRL/*lpr* mice as the primary animal model of lupus. MRL/*lpr* mice develop autoimmune disease resembling lupus caused by mutation (Fas*^lpr^*) that promotes survival of self-reactive lymphocytes, and a strain background (MRL/MpJ) predisposed to autoimmunity. MRL/*lpr* mice are characterized by massive lymphadenopathy associated with aberrant T cells, arthritis, and immune complex glomerulonephritis. Among aberrant T cells of MRL/*lpr* mice, double-negative (DN) T cells, defined by the absence of CD4 and CD8, are the major pathogenic immune cell subset and have the ability to produce proinflammatory cytokines like IFN-γ and IL-17 which has been linked to lupus pathogenesis both human and mice (16). We indicated for the first time that MDSCs, especially PD-L1 expressing MDSCs, significantly decrease the DN T cell populations in MRL/*lpr* mice (**Figure 5A**).

Eventually, we checked MDSCs population in lupus patients. The population of MDSCs were significantly increased in peripheral blood of lupus patients compared to healthy controls (**Figures 6A, B**). These result are consistent with the previous reports (7, 8). Interestingly, the populations of MDSCs expressing immunoregulatory molecules including PD-L1, arginase-1, and IL-10 were significantly decreased in lupus patients (**Figure 6C**). MDSCs like cells were located in kidney tissues of patients with lupus nephritis but not in healthy kidney area of patients with renal cell carcinoma (**Figure 6D**).

In conclusion, the present study indicates that MDSCs from lupus could be dysfunctional and PD-L1 expressing MDSCs have more potent immunoregualtory activity *in vitro* and ameliorate autoimmunity and kidney damage more profoundly in two murine models of lupus. These findings suggest PD-L1 expressing MDSCs with potent immunosuppressive activity be a promising therapeutic strategy targeting systemic autoimmune diseases.

## Data Availability Statement

The raw data supporting the conclusions of this article will be made available by the authors, without undue reservation.

## Ethics Statement

The studies involving human participants were reviewed and approved by by the Institutional Review Board of Seoul St. Mary’s Hospital of the Catholic University of Korea. The patients/participants provided their written informed consent to participate in this study.

The animal study was reviewed and approved by the Animal Care and Use Committee of the Catholic University of Korea.

## Author Contributions

M-JP designed the study, performed experiments, analyzed data, and drafted the manuscript. J-AB, JC, SJ and D-SK performed experiments and analyzed data. S-HP analyzed data, revised critically the final version. M-LC and S-KK designed and organized research and edited the manuscript. All authors contributed to the article and approved the submitted version.

## Funding

This work was supported by the National Research Foundation of Korea (NRF) grant funded by the Korea government (MSIT) (No. NRF-2018R1A2A2A05018848). This research was supported by a grant of the Korea Health Technology R&D Project through the Korea Health Industry Development Institute (KHIDI), funded by the Ministry of Health and Welfare, Republic of Korea (grant number HI20C1496).

## Conflict of Interest

The authors declare that the research was conducted in the absence of any commercial or financial relationships that could be construed as a potential conflict of interest.
