# Supplementary figures and images for: Programmed Death-Ligand 1 Expression Potentiates the Immune Modulatory Function Of Myeloid-Derived Suppressor Cells in Systemic Lupus Erythematosus
Source: Front Immunol. 2021 Apr 27;12:606024. doi: 10.3389/fimmu.2021.606024 (PMC8110929; doi:10.3389/fimmu.2021.606024)

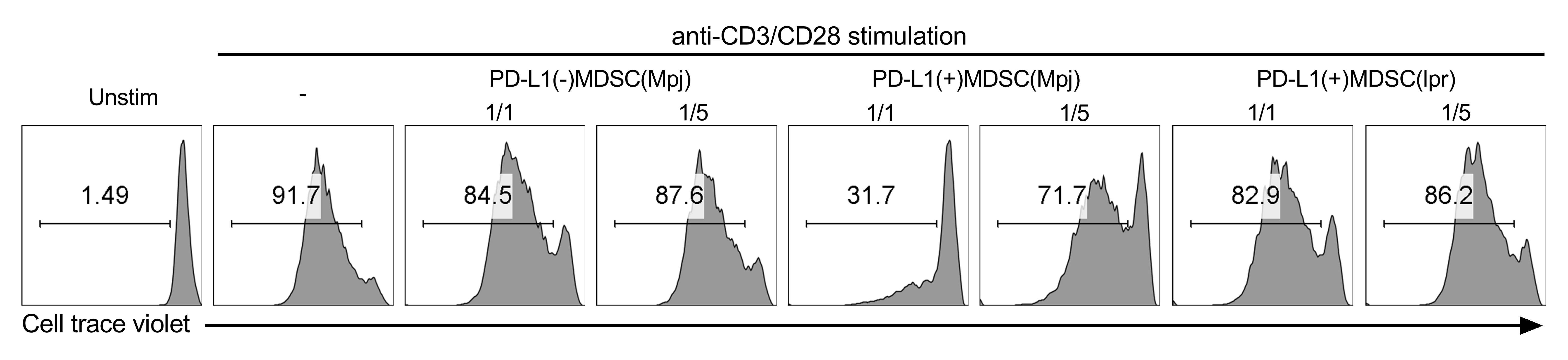

Supplement: Supplementary file 1 [file Image_1.tif]

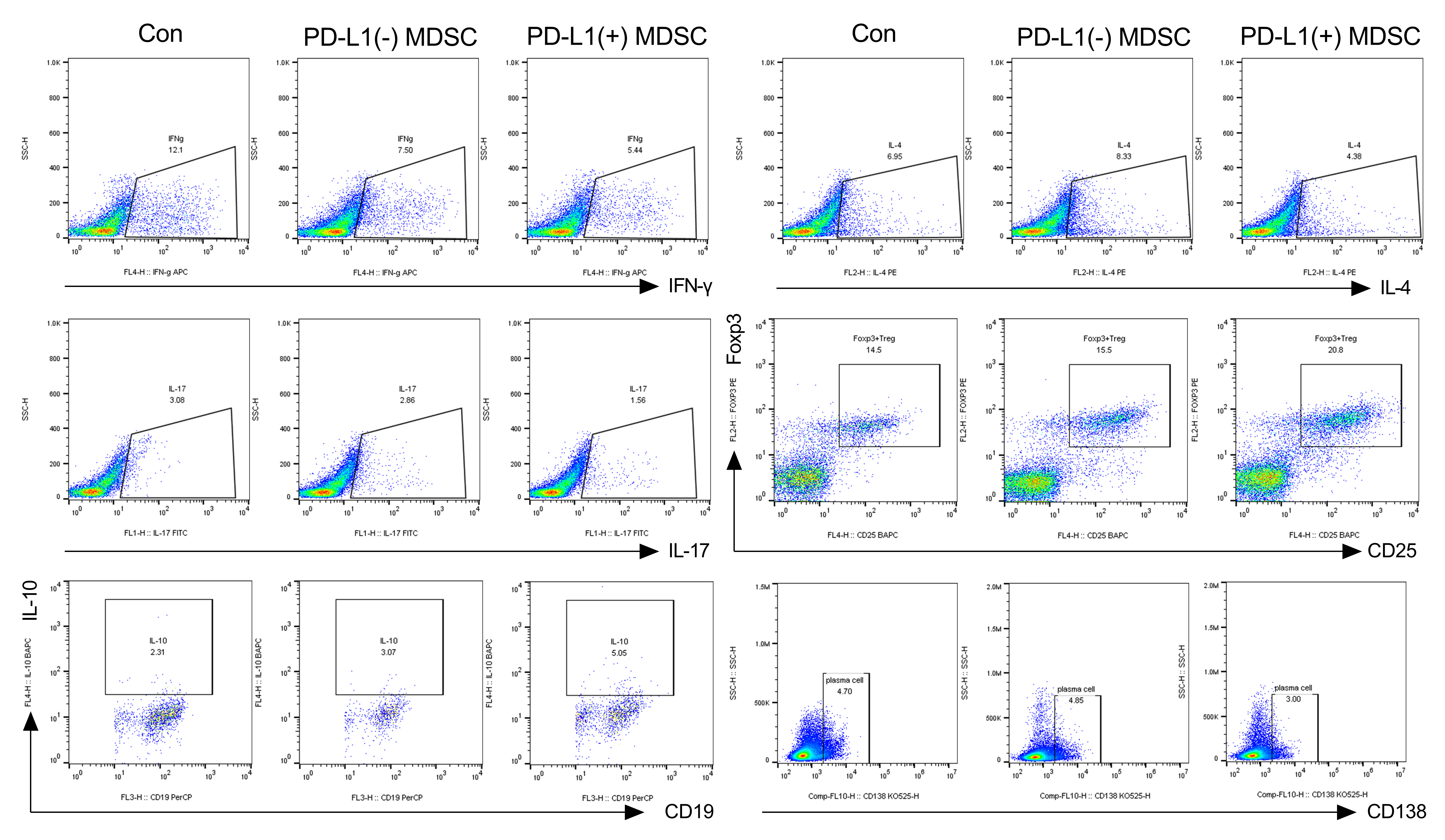

Supplement: Supplementary file 2 [file Image_2.tif]
