# Supplementary material for: Programmed Death-Ligand 1 Expression Potentiates the Immune Modulatory Function Of Myeloid-Derived Suppressor Cells in Systemic Lupus Erythematosus
Source: Front Immunol. 2021 Apr 27;12:606024. doi: 10.3389/fimmu.2021.606024 (PMC8110929; doi:10.3389/fimmu.2021.606024)
Supplement: Supplementary file 3 [file Table_1.docx]

**Table S1. Mouse primers used for real-time PCR**

|  | **Forward** | **Reverse** |
| --- | --- | --- |
| Arginase-1 | CAGAAGAATGGAAGAGTCAG | CAGATATGCAGGGAGTCACC |
| IDO | GACGGACTGAGAGGACACAG | GGCAGCACCTTTCGAACATC |
| PD-L1 | AAAGTCAATGCCCCATACCG | TTCTCTTCCCACTCACGGGT |
| IL-10 | GGCCCAGAAATCAAGGAGCA | AGAAATCGATGACAGCGCCT |
| iNOS | CAGCTGGGCTGTACAAACCTT | CATTGGAAGTGAAGCGTTTCG |
| IL-6 | AACGATGATGCACTTGCAGAAA | TCTGAAGGACTCTGGCTTTGTC |
| TNF-a | AAGCCTGTAGCCCAC GTCGTA | GGCACCACTAGTTGGTTGTCTTTG |
| VEGF | GTCCGATTGAGACCCTGGTG | ATCCGCATGATCTGCATGGT |
| TGFβ | GCCTGAGTGGCTGTCTTTTGA | CACAAGAGCAGTGAGCGCTGAA |
| β-actin | GTACGACCAGAGGCATACAGG | GATGACGATATCGCTGCGCTG |
